# Screening of key genes related to the prognosis of mouse sepsis

**DOI:** 10.1042/BSR20202649

**Published:** 2020-10-30

**Authors:** Muhu Chen, Xue Chen, Yingchun Hu, Xianfu Cai

**Affiliations:** Department of Emergency Medicine, Affiliated Hospital of Southwest Medical University, Luzhou, China

**Keywords:** Sepsis, Prognosis, PPI co-expression, RNA-seq

## Abstract

Sepsis is a common clinical disease with high mortality, and patients with sepsis have varied prognoses. Researchers need to explore the underlying mechanisms that determine the prognosis of sepsis. Hence, a mouse model was used to evaluate new potential prognostic markers of sepsis. Mice were randomly divided into low-dose group (*n*=3, lipopolysaccharides [LPS], 20 mg/kg) and high-dose group (*n*=3; LPS, 40 mg/kg). Total RNA was extracted from the peripheral blood of mice, and samples were then subjected to RNA sequencing. When complete data were normalized, the high-dose group and low-dose group were screened for differentially expressed genes (DEGs, log2FC ≥ 1 and q value ≤ 0.05). DEGs were analyzed by gene ontology enrichment, and potential core genes were screened using protein–protein interaction (PPI) network and weighted gene co-expression network analysis (WGCNA). Moreover, the survival data in GSE65682 were used to observe the correlation between core genes and prognosis. A total of 967 DEGs were identified in the low-dose group, of which 390 were up-regulated and 577 were down-regulated. These genes were mainly enriched in white blood cell activation, lymphocyte activation, immune system response etc. *LCK, ZAP70, ITK, CD247*, and *DOCK2* were found at the core of PPI network, while WGCNA found that interferon-inducible protein 35 (*IFI35*), *ITGB3*, and mediator complex subunit 25 (*MED25*) may be potential core genes. It was demonstrated that *CD247, DOCK2, IFI35, ITK*, and *LCK* core genes were positively correlated with prognosis based on GSE65682. CD247, DOCK2, IFI35, ITK, LCK, and MED25 might be important targets affecting the prognosis of sepsis.

## Introduction

Sepsis is a common critical clinical illness with high mortality, and data show that its incidence is approximately 19 million patients per year in the United States [1]. The latest guidelines defined sepsis as an infection that causes dysfunctions in more than two organs [2,3]. The current clinical treatment for sepsis consists of anti-infection, fluid replacement, and organ function maintenance. With regard to its pathogenesis, current researchers have discovered that sepsis is related to inflammatory storms [4,5]. Patients with sepsis may survive or die even when receiving the same treatment. However, the key factors and mechanisms that determine its prognosis remain unclear. The present study intends to simulate the different prognoses of sepsis through intraperitoneal injection of different concentrations of lipopolysaccharides (LPS) in mice and sequence its peripheral blood cells to explore potential core factors in order to screen out the key targets that determine its prognosis.

## Materials and methods

The experimental mice were obtained from the Experimental Animal Center of Southwest Medical University. Male Kunming mice aged 8–12 weeks, weighing 25–30 g were selected for the present study. The mice were housed in the Animal Laboratory of the Central Laboratory of Southwest Medical University, with a 12-h day–night cycle and an ambient temperature of 24 ± 2°C. Moreover, mice were raised in an ordinary rearing environment with free access to water and food. The experiments on animals began after 5 days of acclimatization to the environment. All animal experiments were approved by the Animal Ethics Association of Southwest Medical University. LPS was purchased from the American Sigma Inc (Fungus: O55: B5).

### Grouping and modeling

The pre-experimental mice were randomly divided into four groups, each with ten mice, which summed up to a total of 40 mice. The mice were fasted for 10 h prior to the experiment and resumed eating 1 h after modeling. The mice from each group were administered with intraperitoneal injection of LPS and equal dose of PBS solution with drug concentrations of 10, 20, 40, and 50 mg/kg. Thereafter, their body temperature and activity status were measured 0, 2, 4, 8, 12, 24 h, 2, 3, 4, 5, and 7 days after injection, and the survival curve was plotted to determine the best modeling conditions. In the observation study, some mice died within 7 days due to the LPS intraperitoneal injection; those surviving over 7 days were killed by cervical spondylectomy. In the formal experiment, the mice were randomly divided into two groups: the survival group (*n*=3) and the death group (*n*=3). The survival group (low-dose group) received intraperitoneal injection of an equal volume of PBS solution and 20 mg/kg LPS, while the death group (high-dose group) received intraperitoneal injection of 40 mg/kg LPS. The body temperature and activity status of each group of mice were measured during the above time points. When the body temperature of the mouse in the death group reached 23°C (dying state), the blood from the eyeball was taken. At the same time point, one mouse from the survival group was randomly selected to have its eyeball blood taken, and the sample was treated with EDTA-2K for anticoagulation. During collection of blood samples, the survival group mice died of massive hemorrhage due to eyeball enucleation.

### Sample sequencing

In accordance with the instructions of Invitrogen, the TRIzol method was used to extract the total RNA from whole blood, and DNase I (TaKaRa) was used to eliminate the genomic DNA. The RNA quality was then measured by 2100 Bioanalyzer (Agilent) and then quantified using ND-2000 (NanoDrop Technologies). Only high-quality RNA samples (OD_260/280_, 1.8–2.2; OD_260/230_ ≥ 2.0; RIN, ≥ 6.5; 28S, 18S ≥ 1.0, > 2 μg) were used to construct sequencing library. One microgram of the qualified test sample was taken. The test samples were prepared, the library was built, and the RNA-seq was sequenced according to the Illumina TruSeq™ RNA sample manual.

### Differential gene screening

All clean sequencing data were compared using the GenBank mouse genome database, and subsequently, gene annotation was performed. To ensure the accuracy of gene expression levels, the fragments per kilobase of exon per million (FPKM) was employed to normalize gene expression levels. By performing log-uniform analysis on differentially expressed genes (DEGs), the differential genes in the survival and death groups caused by LPS were screened (screening conditions: log2FC ≥ 1 and q value ≤ 0.05). Moreover, data visualization was carried out through integrated Differential Expression and Pathway analysis (iDEP) (http://bioinformatics.sdstate.edu/idep/) network tool based on R language [6].

### GO analysis

GO analysis is a typical analysis method for big gene data. It can perform global analysis from three levels, BP, MF, or CC, which is beneficial to the classification and screening of batch genes in the later stage. In the present study, GO enrichment was performed on up-regulated and down-regulated genes according to different gene expression trends.

### Protein–protein interaction analysis

The protein–protein interaction (PPI) network is a common method used in screening potential core genes, and the STRING (https://string-db.org/cgi/input.pl?) online network tool is a popular tool for PPI analysis [7]. The basic principle is to connect proteins that directly and physically interact with each other. In theory, the more core factor functions, the more connections and the closer to the core area. Considering the reliability of the data, the present study screened all experimentally verified results. In view of the integrity of the network, the correlation strength coefficient was set to 0.3. Subsequently, through the online analysis tool, the functions of interest in the present study were screened out, and core genes functions were reviewed.

### Co-expression analysis

In order to prevent the PPI method from being missed, the weighted gene co-expression network analysis (WGCNA) co-expression method was adopted for core gene screening [8]. The purpose of co-expression analysis is to screen out possible key factors based on the common trend of expression values [9]. WGCNA co-expression analysis is divided into different modules according to different expression situations. This method can reasonably speculate the possible functions of genes that are less studied. For core genes screened by PPI and WGCNA, heat maps were used to establish their expression in different groups.

### Survival curve of core genes

Clinical data are the foundation of scientific research. In order to better screen genes related to prognosis and verify the relationship between core genes and prognosis, human sepsis data GSE65682 [10] were used. GSE65682 was a big data study on genes in the peripheral blood of patients with sepsis to identify the biomarkers of sepsis and other diseases. It recorded the peripheral blood cell gene data and clinical data of 479 patients with sepsis as well as the 28-day survival data. The specific genes of all 479 patients were sequenced from the low-expression group to the high-expression group. The first half (*n*=239) was the low-expression group, while the last half (*n*=240) was the high-expression group. As regarding GSE65682 data, GraphPad Prism 7.0 was adopted to plot the survival curve of the above core genes. Log-rank (Mantel–Cox) test was used to identify statistical differences between the two groups of data.

## Results

### Establishment of sepsis survival and death model

**Figure 1:**
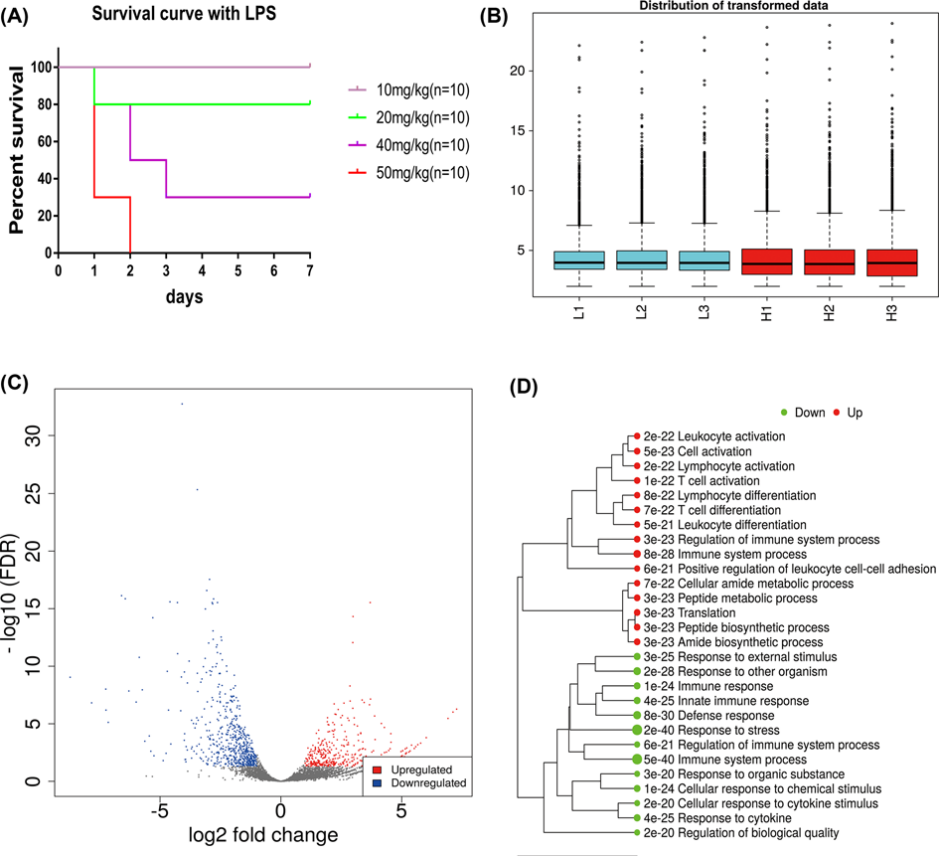
Establishment of LPS model of sepsis in mice and homogenization of blood RNA sequencing data (**A**) Establishment of sepsis model in mice. Different concentrations of LPS were used for intraperitoneal injection. Within 7 days, no mice died in the 10 mg/kg group (simulated SIRS); 20% died in the 20 mg/kg group (survival group); 70% died in the 40 mg/kg group (death group); and 100% died in the 50 mg/kg group (super dose group) within 2 days. (**B**) Box chart after the homogenization of sequencing data of the low-dose group (survival group) and the high-dose group (death group). (**C**) Volcanic map results of the differential gene analysis between the two groups (survival group and death group). The vertical coordinate is the negative logarithm of FDR value (the base number is 10); the horizontal coordinate is fold change: the red on the right is the up-regulated gene, and the blue on the left is the down-regulated gene. (**D**) Functional annotation of up-regulated and down-regulated DEGs.

After an intraperitoneal injection of different concentrations of LPS, the mortality of mice has shown to be dose-dependent. In general, the higher the concentration, the higher the mortality rate (Figure 1A). Furthermore, the overall survival rate of mice in the 20 mg/kg group was 80%, while the mice in the 50 mg/kg group almost died in 48 h. In order to better simulate the clinical situation of sepsis, we chose 20 mg/kg as the survival group and 40 mg/kg as the death group for follow-up research. All survival mice in the low-dose group showed hypomotility, appetite loss, yellowing hair, and other inflammatory manifestations similar to sepsis.

### Identification of differential genes

After sequencing and gene annotation of peripheral blood cells of mice, the gene expression data were logarithmically processed (Figure 1B). As a result, the data were uniform, and the two groups were comparable. In comparison with the high-dose group, a total of 967 DEGs were found in the low-dose group, of which 390 gene expressions were up-regulated, 577 gene expressions were reduced, and DEG distributions were even (Figure 1C).

### GO enrichment analysis

On one hand, GO analysis revealed that the highly expressed genes in the survival group were mainly enriched in leukocytes, T cells, lymphocyte activation and differentiation, immune system processes, positive regulation of leukocyte adhesion, and peptide metabolic processes. On the other hand, genes highly expressed in the death group were mainly enriched in immune response, endogenous immune response, defense response, and response to external stimuli. The enrichment of these functions basically reflected the changes in biological processes in different states, as illustrated in Figure 1D.

### PPI screening of core factors

**Figure 2:**
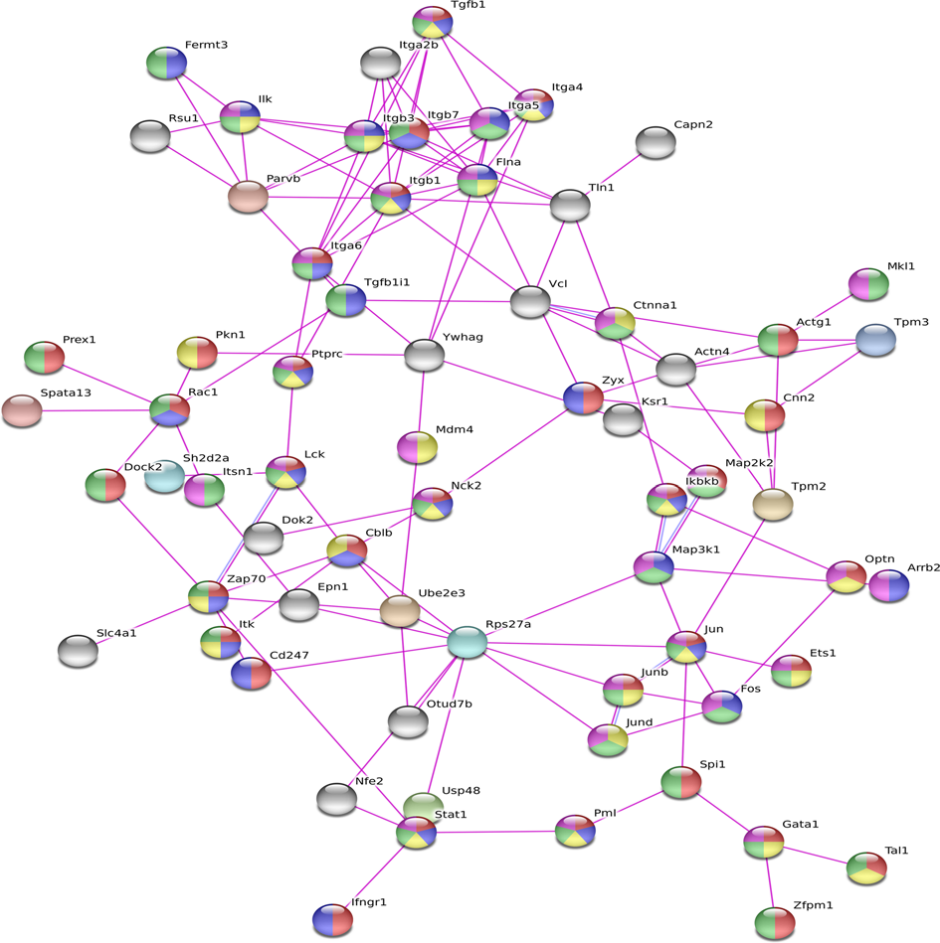
The PPI network of DEGs was constructed using STRING tool; different colors represent different functions: red is the immune system process; blue is the cell surface receptor signaling pathway; yellow is the regulation of cell population proliferation; green is cell differentiation; and violet is the regulation of cell death

In the present study, the PPI network analyzed by the STRING online tool included 67 core proteins, which were closely linked together. The proteins of LCK, ZAP70, JUN, ITK, CD247, DOCK2, and HCK were located at the core of the network, which might be involved in many different functions. In the present study, proteins involved in the immune system, cell surface receptor signaling pathways, cell proliferation regulation, cell differentiation, and cell death regulation were paid more attention, as illustrated in Figure 2.

### Co-expression screening of core genes

**Figure 3:**
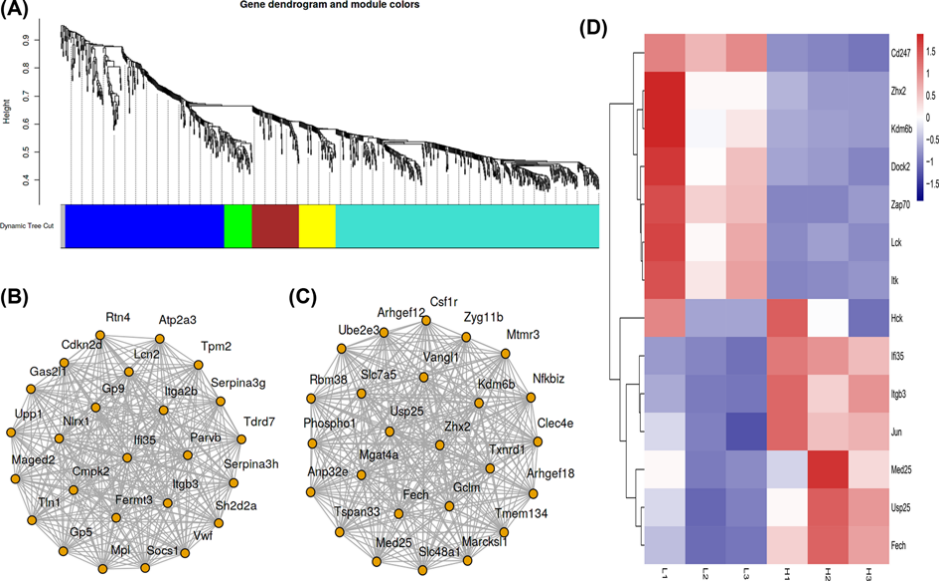
Construction of co-expression network based on WGCNA and screening of core genes (**A**) The module distribution of WGCNA co-expression analysis of DEGs. (**B,C**) Module 1/2 shows the first 25 genes of degree construct network with a correlation coefficient of 0.4. (**D**) Heat-map of core genes screened using PPI and co-expression analysis; red for up-regulated; blue for down-regulated.

**Figure 4:**
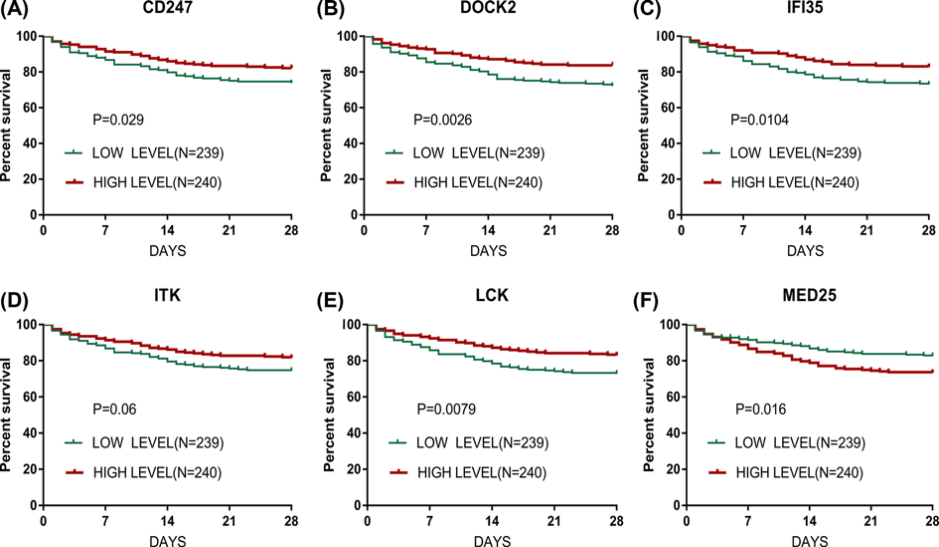
Survival curve of six potential cores in GSE65682 (**A**–**F**): (A) CD247; (B) DOCK2; (C) IFI35; (D) ITK; (E) LCK; (F) MED. Red for high-expression group, green for low-expression group.

Co-expression analysis is a common method of bioinformatics, which supplements the PPI analysis deficiency. We employed the WGCNA analysis method to construct a co-expression module for genes. A total of five modules were screened out in the present study, and two of them were mainly distributed. We visualized the first 25 genes of these two modules and discovered that interferon-inducible protein 35 (*IFI35*), *ITGB3, ZHX2, USP25, FECH, KDM6B*, and mediator complex subunit 25 (*MED25*) might be the potential core genes, as shown in Figure 3A–C. In Figure 4D, we further observed the expression of 14 potential core genes screened by two of the abovementioned methods of PPI and WGCNA. CD247, ZHX2, KDM6B, DOCK2, ZAP70, LCK, and ITK were highly expressed in the survival group, while IFI35, ITGB3, JUN, MED25, USP25, and FECH were highly expressed in the death group.

### Survival curve of core genes

To further explore the correlation between genes, we screened the prognosis of clinical patients, and the genetic data of sepsis peripheral blood cells in GSE65682 were used for survival analysis. Finally, we found that the expression values of CD247, DOCK2, IFI35, and LCK manifested a positive correlation with the prognosis of patients (*P*<0.05), and the difference in the statistics of ITK survival curve was *P*=0.06. The expression value of MED25 demonstrated a negative correlation with the prognosis of patients (*P*<0.05), as shown in Figure 4.

## Discussion

Sepsis is a common critical clinical condition, especially in patients in the ICU. Sepsis involves multidisciplinary and multisystem changes [2]. Clinically, some patients with sepsis died despite strong anti-infection treatment, especially when sepsis is combined with septic shock. In clinical practice, the key factors that determine the prognosis of patients and their underlying mechanisms deserve our in-depth study [11]. In this study, different concentrations of LPS were used to construct the survival and death model of sepsis. Moreover, the mortality was used as the main standard to screen and simulate different prognoses of sepsis. Furthermore, the cecal ligation and perforation (CLP) method is another common model construction method [12]. However, achieving the expected results of CLP during the construction process is challenging. This may be related to the large differences in individual defense responses in the abdominal cavity of mice, especially the omentum.

In the present study, peripheral blood cells were sequenced and screened under bioinformatics technology. A total of 967 DEGs were screened out. Among them, 390 genes were up-regulated and 577 genes were down-regulated in the survival group. Through GO analysis, it was found that gene function enrichment in the survival group was mainly related to the activation and differentiation of white blood cells, lymphocytes, and T cells, which suggested that the body is establishing a second line of defense, or may participate in the establishment of endotoxin tolerance mechanisms. The gene function enrichment in the death group was mainly related to immune response and endogenous defense response, which indicated that the body produces a large number of inflammatory mediators and induces an inflammatory cascade amplification effect. This was consistent with our ‘inflammatory storm’ theory.

In the method of screening core genes, we used PPI and WGCNA to screen the genes together [7–9], as far as possible, to prevent possible missed selection of a single method. From a methodological point of view, the PPI method is more likely to be verified later due to the multiple functions of these core proteins confirmed by a large amount of data in the early stage. Additionally, co-expression will likely select core genes that are less studied, so it is with higher innovation. However, all core genes theoretically screened need to be verified by experiment.

To further increase the positive rate of bioinformatics screening, we included the clinical prognostic data of patients. Verification in the human body will make the conclusion more convincing. The present study screened six potential core genes: *CD247, DOCK2, IFI35, LCK, MED25*, and *ITK*. Among them, *CD247, DOCK2, IFI35, LCK*, and *ITK* showed positive correlation with prognosis, suggesting that they might be beneficial to patient survival. The *MED25* gene was negatively correlated with the prognosis, which indicated that it plays a role in the promotion of death process of patients.

CD247 is a characteristic protein located in the T-cell membrane. Existing research revealed that it is mainly related to immune diseases [13–16], while there is no research on sepsis. Under this circumstance, it could be a potential drug treatment target. LCK is a non-receptor tyrosine protease, which plays a vital role in promoting the maturation and differentiation of T cells and their functions after maturation. Once T cells are stimulated, TCR activates the tyrosine kinase ZAP70, and ZAP70 is phosphorylated and activated by LCK. Thereafter, a large number of signal molecules are absorbed, which ultimately leads to the production of lymphokines [17]. Subsequently, studies have demonstrated that LCK phosphorylation inhibits inflammatory storm via TLR4 signaling pathway [18]. DOCK2 is an atypical guanine exchange factor specifically expressed in hematopoietic cells. It regulates the activation and migration of immune cells by activating Ras-related C3 botulinum toxin substrate (Rac). Moreover, Dock2 plays a significant role in the occurrence and development of various inflammatory diseases [19–22]. IFI35 is an IFN-γ-induced protein that plays an important role in anti-virus-related immune inflammation. Overexpression of IFI35 affects endothelial cell proliferation, migration, and re-endothelialization through the NF-KB pathway [23]. Furthermore, studies have also illustrated that IFI35 exacerbates H5N1 influenza by expressing IL-12p40 homodimer [24]. In addition, ITK and BTK regulate the thermal homeostasis in sepsis in mice through mast cell function. By regulating the activation of NF-KB, phosphatidylinositol-4,5-bisphosphate, 3-kinase/Akt, and p38 signaling pathway, they play a regulatory function in the downstream of mast cell toll-like receptor 4/LPS [25]. On the contrary, enterovirus 71 (EV71) is the main pathogen of hand-foot-mouth disease, and its neurological complications are particularly serious, which is the main cause of death of the disease. EV71, VP1, and human MED25 have a highly expressed common epitope (co-epitope) in the brain stem, which indicates the involvement of MED25 in the occurrence and development of the disease [26].

The present study confirmed the prognostic correlation of the six core genes screened. However, reports on the screened genes and sepsis are limited, which suggests that they may be potential research targets, while their specific functions and mechanisms need to be further studied.

## Abbreviations

CLP: cecal ligation and perforation
DEG: differentially expressed gene
EV71: enterovirus 71
IFI35: interferon-inducible protein 35
LPS: lipopolysaccharide
MED25: mediator complex subunit 25
PPI: protein–protein interaction
WGCNA: weighted gene co-expression network analysis
